# A qualitative reflexive thematic analysis into the experiences of being identified with a *BRCA*1/2 gene alteration: “So many little, little traumas could have been avoided”

**DOI:** 10.1186/s12913-022-08372-w

**Published:** 2022-08-06

**Authors:** Nikolett Zsuzsanna Warner, AnnMarie Groarke

**Affiliations:** School of Psychology, National University of Ireland, Galway, Ireland

**Keywords:** *BRCA*, HBOC, Uncertainty, Qualitative, Thematic Analysis, Informational Needs, Nursing, Experiences

## Abstract

**Background:**

*BRCA*1/2 alterations increase females’ lifetime breast cancer risk to 40 – 90%, ovarian cancer to 10 – 60%, and males’ lifetime prostate cancer risk to ~ 10 – 25%. Psychosocial issues such as heightened distress can, therefore, occur in this population. This study aimed to explore the subjective experiences and needs of the *BRCA*1/2 alteration population in navigating cancer risk reduction measures.

**Method:**

This study aimed to explore the experiences and identify the needs of 18 *BRCA*1/2 alteration carriers, recruited through strategic sampling. A public and patient panel (*N* = 6) collaborated on study development. Data were analysed using reflexive thematic analysis.

**Results:**

Two themes were identified: (i) Healthcare Services as a Burden to Navigate, and (ii) Burden Experienced Through Interactions with Healthcare Professionals. Results indicated uncertainty regarding care pathways, alongside a lack of relevant information. Participants felt unsupported by healthcare professionals, and as though healthcare professionals often perceive them as a burden.

**Conclusions:**

These findings suggest that the quality of interactions in healthcare systems are of relevance to the *BRCA*1/2 alteration population, and that uncertainty surrounding access to services and information is prevalent. The establishment of specialist hereditary cancer clinics could reduce such burden.

**Supplementary Information:**

The online version contains supplementary material available at 10.1186/s12913-022-08372-w.

## Background

Breast cancer genes 1 and 2 (*BRCA*1/2) act as tumour suppressors, assisting in preventing tumorous growths. These genes can become altered, known as *BRCA*1/2 alterations or mutations, significantly increasing the cumulative lifetime risk for numerous cancers. In females, *BRCA*1 and *BRCA*2 alterations lead to a 72% and 69% cumulative lifetime risk of breast cancer, respectively [1], and a cumulative lifetime risk of ovarian cancer of 44% and 17%, respectively [1]. Lifetime breast cancer risk for males with *BRCA*1/2 alterations is approximately 7%, 80 – 100 times that of the general male population [2]. Lifetime prostate cancer risk in males with *BRCA*1 alteration carriers is thought to be similar to that of the general population, but is approximately 20% for those with *BRCA*2 alterations [3]. Some evidence also suggests increased risks of pancreatic and melanoma cancers in both sexes [4]. While estimates vary, it is thought that worldwide, approximately one in 300, to one in 500 people in the general population carry a *BRCA*1/2 alteration [5–7].

### Cancer prevention in *BRCA*1/2 alteration carriers

Females are often advised to undergo prophylactic double mastectomies with optional reconstruction to reduce breast cancer risk, and bilateral prophylactic salpingo-oophorectomies to reduce ovarian cancer risk [8]. These surgeries are usually implemented around the age of 35 – 40, or if the *BRCA*1/2 alteration is identified after 40 years of age, as soon as is feasible, while taking psychological adjustment and family planning into consideration. Cancer worry and the perceived burden of a cancer diagnosis are noted predictors of motivation to undergo prophylactic surgery in this population [9]. If prophylactic surgeries are not deemed favourable, the impacted female may instead undergo intensive breast screening programmes, with the type of screening dependent on the age, and personal and familial history of cancer [8]. There are no trusted screening tools for the early detection of ovarian cancer. Males are not offered prophylactic surgeries but may subscribe to enhanced screening schedules for prostate cancer.

### Psychological impact of *BRCA*1/2

Uncertainty within the context of *BRCA*1/2 alterations has been highlighted as a potential stressor in this population. This encompasses uncertainty around the future of children and personal medical uncertainty. For females, it also raises issues surrounding femininity and sexuality [10, 11]. The information needs associated with this uncertainty are overlooked. A lack of easily accessible, clear, relevant health information can lead to uncertainty [12], which if not managed appropriately, can lead to distress [13, 14] and ill physical and psychological health [15, 16]. *BRCA*1/2 alteration carriers in this state of uncertainty undergo cognitive appraisals which can lead to the development of management strategies, such as information seeking behaviour [17, 18].

### Theoretical understandings of uncertainty

Theoretical explanations of uncertainty include conceptual approaches that focus on certain stages of an individual’s illness trajectory. The Uncertainty in Illness [19] theory states that uncertainty acts as a cognitive stressor for individuals diagnosed with a chronic illness. This theory states that uncertainty can occur where illnesses are “ambiguous, complex, and unpredictable” [19] – all factors present in hereditary cancer conditions, and previously has been employed to explore experiences of uncertainty in cancer populations [20]. If the individual experiencing uncertainty has access to relevant information, they can be more engaged with decision-making – which is vital to managing risk in hereditary cancer populations [20].

A life-long approach to explaining uncertainty in hereditary cancer populations has also been proposed [21]. Uncertainty is considered a continuous component of the *BRCA*1/2 alteration experience, that can change over time. This can influence health management behaviour, which supports health-promoting or health-inhibiting risk appraisals, subsequently affecting decision-making [21]. This approach suggests that the management of uncertainty needs to be focused on the stage at which individuals experience their uncertainty, such as by taking into account an individual’s age (and therefore the stage of risk management; 23).

### Informational needs

*BRCA*1/2 alteration carriers require clear information on their cancer risks and on the options available to reduce these [22, 23]. This need is often met by charitable organisations, rather than healthcare professionals or services [22]. The provision of information about *BRCA*1/2 alterations has been perceived by the *BRCA*1/2 population to be a positive occurrence, with past qualitative research highlighting that knowledge about the condition is viewed as an advantage [24]. Information gaps are noted between healthcare professionals providing care to those with a *BRCA*1/2 alteration, and those at risk of carrying a *BRCA*1/2 alteration [25].

### Context

This current article is nested within a larger doctoral research project, which aims to study the experiences and needs of the *BRCA*1/2 alteration population (for earlier studies from this project, see: 26). This paper reports on the experiences and needs of the *BRCA*1/2 alteration population navigating cancer risk reduction measures. Further themes were noted in regard to familial stressors and coping with issues external to the healthcare system, however, these are considered beyond the scope of the present article. The majority of research on the topic has been conducted in Israel and the United States. Cultural context has been highlighted as an important factor when understanding experiences, needs and choice of preventative measures in the *BRCA*1/2 alteration population [27, 28].

The current study took place in Ireland, where the healthcare system operates on a tiered basis – care is available through a public or private system. Criticisms exist concerning the waiting lists in the Irish public sector, which in 2022 saw 850 people waiting to see a genetic counsellor, and 1,900 to see a consultant clinical geneticist [29]. The private sector is therefore often relied upon, allowing preferential access [30, 31]. This can cause uncertainty and distress to those trying to access services [32]. Cases of potentially preventable cancer in Ireland have been reported due to prolonged waits for clinical genetic services [33]. This system draws parallels to issues faced internationally, such as in Singapore, Hong Kong and the United States, where individuals often turn to the private system to gain access to timely care.

There is currently no designated specialist service in Ireland for those impacted by a hereditary cancer condition, and often individuals with *BRCA*1/2 alterations are referred to symptomatic oncology units, regardless of their cancer status. A recent report conducted on Irish cancer genetic services noted that one in seven individuals trying to access cancer services found it difficult to obtain information pertinent to their situation [32]. This research aimed to gain insight into the experience of individuals with a *BRCA*1/2 alteration in the Irish healthcare setting.

### Public and patient involvement

Public and patient involvement (PPI) was consistently included throughout this research. The inclusion of stakeholders of any target population can assist in bridging the research-implementation gap through the co-creation of knowledge between the researcher and those being researched [34, 35]. As such, a PPI panel of six individuals with *BRCA*1/2 alterations were involved in this study. Of note is that the lead author carries a *BRCA*1 alteration, thus furthering the involvement of stakeholders in this research. The key aim of including the PPI panel was to ensure that the research question was pertinent to the population under study.

## Method

### Design/qualitative approach

Interviews were analysed using reflexive thematic analysis (TA; [36]), which provides an interpretation of collected data while acknowledging the subjectivity of the researchers’ perspectives [37]. Analysis was conducted through a constructivist paradigm [38]. This is not to say that the analysis was conducted without regard for theoretical considerations – rather, the aforementioned epistemological and paradigm assumptions formed the theoretical backbone through which this research was conducted [38]. In this regard, meaning creation was accepted to have contextual influences, whereby researcher subjectivity was deemed to benefit the analytical process – the researcher was considered in this form of TA to play an active role in knowledge production [37]. The themes reported on acted as the final outcome of the coding and theme development process [38]. Only the lead author coded the transcripts.

### PPI

Contributions from the PPI panel to this study are reported using the GRIPP-2-SF checklist (39, see supplementary table 2). The PPI panel informed the development of the research questions through discussion of the main issues currently faced by the population in the Irish healthcare system, and in broader terms. The recruitment materials and interview schedule were developed by the researcher and reviewed by the panel, with feedback collated and applied. One panel member took part in piloting the interview schedule, which consisted of open-ended questions, to see whether any were deemed inappropriate or irrelevant when administered verbally. This interview was not recorded or included in the analysis. The PPI panel did not take part in transcribing or coding of interviews, due to the sensitive and identifiable nature of the data collected.

### Recruitment and sample size

Participants were required to be over 18, carry a known *BRCA*1/2 alteration, and have undergone genetic testing in Ireland. No restriction was applied on the sex of participants, time since genetic test, and preventative measures undergone (or not). As data saturation is not deemed an appropriate concept to ascertain sample size within reflexive thematic analysis [40], the final sample was contingent on the supposed quality of data collected to address the aims of this study – not when no new information was thought to be collected [40].

### Reflexivity and positionality

An important aspect of conducting reflexive qualitative research is to note the researchers’ backgrounds when approaching the study [38]. With regards to the understanding of the phenomenon at hand, the lead author, NW, has a *BRCA*1 alteration and has worked in advocacy and peer support for the *BRCA*1/2 alteration population for numerous years. The lead author conducted all the interviews. This further enhances the understanding of contextual factors discussed in the results [41]. Participants were aware of the researcher’s genetic status when taking part in the interviews but were informed that it was their own experiences that were of relevance to the research. To ensure that this influence was acknowledged during the research process, the lead author undertook regular reflexive practice. The lead author’s doctoral supervisor (AMG), a chartered health psychologist with subject expertise in the field of qualitative research in psycho-oncology, further provided insight into conducting reflexive thematic analysis.

### Data collection

Ethical approval was granted from the Research Ethics Committee from the National University of Ireland, Galway (REF: 19-Aug-23). Participants were recruited through closed Facebook groups and from an article in a national newspaper. Individuals expressed their interest by contacting the researcher at their institutional email address. A participant information sheet and consent form were then sent via Microsoft Forms. In the case of one individual who was not computer literate, contact was made by phone and the relevant forms were sent by post, with stamped return envelope provided. Interviews were conducted over the phone, audio-recorded using an OLYMPUS digital voice recorder (WS-852), and then transcribed using Otter.ai software. Interviews were planned to be conducted in person or by phone. As participants resided throughout the Republic of Ireland, it was deemed practical to offer participants the option to conduct interviews over the phone. The option of in-person interviews was then removed due to the COVID19 pandemic, and, therefore, only phone calls were utilised for interviews. The interview guide is presented in Table 1 below. Interview transcripts were anonymised and imported into NVIVO12.

**Table 1:** Interview Guide

1	Why don’t we begin by you telling me a little bit about yourself, and how you found out about your *BRCA*1/2 alteration. What I’d really like to know here is about how you found out about having a *BRCA*1/2 alteration, and what that was like for you
2	Overall, how do you think you coped with learning about your *BRCA* alteration? **PROMPT**: Do you think it affected your day-to-day life?
3	Did you go to seek more information and support? If yes: Where?
4	Broadly, can you tell me about how your relatives feel about *BRCA*? Do you think any of your relationships with your family were affected by the news?
5	Do you talk openly about it within your family?
6	Were any of your relationships with your friends, or partner if you had one at the time, affected by the news?
7	Did you feel like you had enough support to cope with your *BRCA*1/2 alteration?
8	If the participant has children: So the next thing I’d be interested in, is if you can tell me about talking with your kids. Do you discuss your *BRCA*1/2 alteration with your children?
9	If yes to Q. 8: Did you feel prepared to do this? **PROMPT**: Did you seek information or support before bringing up the topic?
10	I think this is a nice question to end on: what advice would you give to someone that has just found out that they have a *BRCA*1/2 alteration?

### Data analysis

While the procedure undertaken is described in a linear manner, this process was iterative in nature, as per Braun and Clarke [36]. Data familiarisation was undertaken, wherein the researcher became immersed in the data through casual note taking and rereading transcripts. Codes were generated by attaching clear labels to sections of text. A bottom-up, inductive perspective was applied, allowing for themes to be developed from the data, rather than relying on predefined themes or a set theoretical approach. The coding phase focused on data in a latent nature, allowing for deeper levels of meaning to be created [37]. Candidate themes were developed, allowing for clear shared meanings to be identified and moulded from codes. Further to this, candidate themes were related back to the research aims through a process of thematic mapping [37]. Finally, themes were revised and defined in detail to allow for the merging of similar candidate themes, allowing clarity in themes included at the final stage.

### Trustworthiness

The Standards for Reporting Qualitative Research (42, see supplementary table 1) were utilised in reporting the study. Throughout this process, the researcher took part in reflexive practice in the form of a reflexive journal following the completion of each interview. Furthermore, peer support and supervision from the lead author’s doctoral supervisor was an important aspect of ensuring that subjectivity was acknowledged, allowing for a considerate analysis and interpretation of data [43]. This was conducted to ensure that theoretical assumptions were an active element of the analysis.

## Results

Eighteen participants took part in interviews, which took on average 56 min (range: 27 – 80 min). The majority of participants were female (88.89%, *n* = 16), and most had children (83%, *n* = 15). Further participant characteristics are listed in Table 2. There were two themes identified in the data, (i) Healthcare Services as a Burden to Navigate, and (ii) Burden Experienced Through Interactions with Healthcare Professionals (see supplementary Fig. 1).

**Table 2:** Participant Characteristics (*N* = 18)

Characteristic		**Number (%)**
Sex		
	Male	2
	Female	16
Age		
	18–25	0
	26–35	3
	36–45	5
	46–55	7
	56–65	2
	66–75	0
	76–85	0
	86–95	1
†Ethnicity		
	White Irish	16
	White Other	1
	Other	0
Children		
	Yes	15
	No	3

^†^ Missing data for one participant

### Public and patient involvement

The PPI panel suggested that it would be beneficial to include males in the study, as the initial inclusion criteria that were proposed by the first author stipulated that only female participants would be recruited. This change was decided upon as males are often omitted from research on *BRCA*1/2 alterations, as it is often perceived as a ‘female only’ issue [44]. As such, males were also recruited for this study. The interview schedule was also refined to shorten interview questions, as per feedback from the piloting of the interview. The phrasing of certain questions were changed, to ensure the language used was inclusionary and understandable. An example of this was stating “Broadly, can you tell me about how your relatives feel about *BRCA*?”, followed by “Do you think any of your relationships with your family were affected by the news?”, rather than “Were your relationships affected by your *BRCA* mutation diagnosis?”, which was initially included in the interview guide. Further commentary on the input from PPI on language deemed acceptable for the *BRCA*1/2 population has been detailed elsewhere [26].

### Theme one: healthcare services as a burden to navigate

This theme was comprised of two subthemes. Overall, the theme noted that a common uncertainty was experienced through engagement with services, wherein there was significant difficulty with navigating the healthcare system. Participants reported feeling as though they were not welcomed when describing their interactions with the healthcare system. The first subtheme considered issues around the inaccessibility of services, be it through the non-existence of a service, or a lack of availability. A second subtheme denoted the inappropriateness of services available, in that they were not specialised to cater for individuals impacted by hereditary cancer conditions.

### Subtheme one: healthcare services as largely inaccessible

The healthcare system has a key role in the life of *BRCA*1/2 alteration carriers. Engagement with services and service providers played an important function in how participants coped with their heightened cancer risk. The perception of being a burden on the healthcare system was stated by multiple participants. The noted inaccessibility of these services was experienced through a lack of available information to assist with decision-making, and also through a lack of service availability. Both of these concepts overlapped to produce the overall experience of an inaccessible service for individuals with a *BRCA*1/2 alteration.

Of note was a clear distinction between the experiences of males and females. The main discussion here focuses predominantly on the experiences of the female participants in this study. The male participants (*n* = 2) seemed satisfied with the level of information and services received. As one stated:

You are actually very well looked after when you're in the system… it's getting into the system over here is the hard part… that's just another tool they have isn't it, like? They know you’re *BRCA*1, and they will probably be more careful on checks on that front as well.

This sentiment was reiterated, whereby another male participant stated, “I just thought, it was a good experience, in the sense that we felt that we had got very good, eh, professional advice”. In contrast, females noted issues around accessing services and pertinent information, “…I think I did look at stuff in the NHS actually. But you’re reading it going, ‘awh Jesus like, this is in England’”. This is repeated by others, who expressed frustration at wanting to source information but meeting roadblocks in trying to do so, “we’re the people who are willing to go and take those steps and need help and guidance and there is nowhere to turn to get it”. Furthermore, females were not well informed as to the fact that prophylactic surgeries are funded through the public system, as demonstrated by a participant’s initial uncertainty around being able to finance her risk reduction measures:

I don't have a medical card and I don't have insurance… As soon as I got the phone call… I said to [partner], it was later on that night and started crying. I said, "How the hell am I gonna afford this?".

This is further stressed as an issue, whereby a participant noted the uncertainty around the surgical route and how to fund it, should any complications occur, “I had my health insurance, ehm, and I know I could've, I know the genetic centre were covering it, but I said, if I've any complications, I'm screwed. I'd never be able to keep a roof over my head”. Additional issues within the two-tier system were mentioned, wherein individuals were often unaware of the options available to them within the private sector, as one participant stated that her family member “was not given any information then at all about private testing options”. As further highlighted, this negative experience with wait times was further exacerbated due to the lack of information provided to her on alternative routes to gain access to the required services:

But I didn’t realise that I probably could have gotten private for that with [health insurance company]… I was thinking, God, if I had known that earlier, I could have known earlier and I could have maybe avoided the issues that I had, the ovarian issue that I had… that could have potentially been avoided.

One participant further commented on this uncertainty around where to access relevant services, “I have no idea where she should go for counseling or who to go to” when discussing how her daughter could access genetic testing. This demonstrated the interaction between the lack of available services and information. Participants noted how difficult it was to access certain services, which often made them feel like they were presenting as a burden to the healthcare system. As one participant stated:

The decisions are hard enough, but then when the decisions are made morechallenging because the system cannot provide – even when you can make the decision, they can’t implement your decision.

Prolonged wait times seemed ingrained within the country’s two-tier system, “it’s all going privately because if you’re public, well, Jesus, the wait times are ridiculous”. Another participant felt abandoned by the system when trying to seek information and support: “I definitely needed something and I was kind of met with a wall… I had nowhere to go for help”. As one *BRCA*1/2 alteration carrier detailed her experience, having resorted to travelling out of the country to access the services she needed before undergoing her salpingo-oophorectomy, “I brought myself off to [England] to see a hormone specialist”. As another participant depicted, the lack of a designated service meant that when she contacted the head of a respected cancer charity for support, her needs were not met:

And [head of cancer charity] said, we have no resources to support people in your situation, who are informed, who are trying to navigate what the right course of action is… We can only deal with people with an active cancer diagnosis. So basically, there was nowhere to turn.

### Subtheme two: health care services as inappropriate

It is important to note that not only was the access to initial services perceived as lacking, but that minimal continuity of care was reported. The participants felt that there was a lack of coordination among the information provided:

The ﻿only appointment I’ve had so far was in the breast clinic… I asked about the salpingo-oophorectomy, down the line, and I was kinda told ‘Oh you can ask your gynaecologist when you’re at your next maternity appointments’… I just haven’t felt that there’s a strong line of information there.

Similar experiences were noted by others, whereby one participant recounted how she attended an appointment to discuss pancreatic screening, and stated, “I’m not joking, like he was there looking up guidelines about *BRCA* online, sitting there in front of me… it was the biggest waste of an hour of my life”. These issues are noted again by another participant, who recalled her decision-making process around reconstruction following her preventative bilateral mastectomy:

It ﻿was only afterwards, he was offering me the two types of surgery he did, not necessarily the two he thought would be best for me… even as an educated person, who is empowered and whatever else – took me about six weeks to think ‘Oh hang on, I could actually change doctor here’.

This was also experienced by others, “It's like a menu, a menu of mastectomies. I don't really know what to get”. This lack of specialist information created a burden, wherein the individual was left with uncertainty about how to manage their risk, rather than receiving support from the healthcare system. This is further touched on by another participant, who stated, “You could be talking to someone and they could be giving you totally bad advice”. Another individual discussed her experience of having to push for information when managing early onset menopause following her salpingo-oophorectomy, and noted that “nobody mentioned anything, Vitamin D, follow-up, cardiovascular risk – anything” and that “even to this date, and I’m pushing for it”. This lack of assistance was often felt by the participants. Being disregarded by the healthcare system increased distress, “you become very accustomed to being the person with the most information in the room – which is very disconcerting when you’re the patient”. One participant discussed how she received minimal clarity from her general practitioner (GP), and said, “I got very little information – I don't even think my own GP – I’d say I know more about it than she does”. As one participant summarised, “we’re going to these people who are supposed to be the experts, but we’re not walking away with expert advice”. The lack of adequate knowledge and services for those affected by hereditary cancer conditions led to discomfort and uncertainty when attempting to access required services.

Furthermore, the importance of timing in providing information was noted by a participant, who recalled when she was first identified as carrying a *BRCA*1/2 alteration, “I just wasn't in the headspace to deal with everything at the time. So I kind of stepped back from it all”. This is further seen when those who engaged with the healthcare system for screening or preventative surgeries tried to communicate with professionals who were less accustomed to dealing with hereditary cancer conditions. One individual discussed how she found it difficult to be within the screening system, and stated, “I don’t want to have this in front of me”. As another recounted, she found the uncertainty around where to access information difficult:

You kind of don't know who to go to with these questions … like, they crop up, you know, over time. They don't all come within the first week – like it can take – you know, I still come up with new ones now… I'm still like ‘Bloody hell I never asked that’. And you feel like you don't really have anyone to kind of ask.

This highlighted the need to ensure ongoing support in making decisions around risk management.

There are currently no set models of care for hospitals in Ireland on the provision of preventative and screening care for the *BRCA*1/2 alteration population – the uncertainty of being unaffected by cancer, while attending symptomatic oncology services was felt throughout the *BRCA*1/2 alteration population: “You’re sort of going in as a blooming healthy person into a cancer clinic and you’re sort of going, lads, you know, you should just sort this out?”.

One participant noted her worries about the lack of clarity in her healthcare, “do I have faith in the monitoring system? No, no… there’s lots of holes in it, there’s nothing regular”, and stated that there was no one to highlight this concern with “I didn’t have anyone to ask is that normal, should I be giving out, should I be making myself heard?”. Poignantly put by another, if services were more appropriate and specialised, “so many little, little traumas could have been avoided… [hospital] were dealing with my screening but they really had no expertise … it was traumatic, to be honest”.

### Theme two: burden experienced through interactions with healthcare professionals

The second theme represented the influence that healthcare professionals had on individuals’ feelings of being dismissed and is comprised of two subthemes. The first subtheme depicted the lack of understanding among healthcare professionals about the emotional impact of having to make decisions about risk management, which often led to negative interactions. This was expressed through commentary on the lack of awareness and empathy shown by a variety of healthcare professionals. The second subtheme portrayed a disempowerment around decision making, wherein participants discussed how they were regularly dismissed by their healthcare professionals.

### Subtheme One: negative interactions with health care providers

Adding to the burden that individuals perceive that they present in the healthcare system, participants often felt misunderstood by healthcare professionals, as per one participant: “…like doctors, they work in it and it’s grand and all, but I’ve known, I know from my own experience, they don’t understand”. As another participant stated, “there is such a lack of awareness of something like that, life-changing impact… I definitely had to carry a huge amount of it myself”. This is reiterated by other participants, “there wasn’t any support, or there wasn’t anybody that you were told, okay, this is somebody that you can go and speak to now to give you guidance or you know”. Healthcare professionals were often perceived to be dismissive towards individuals with a *BRCA*1/2 alteration, as one participant noted “the attitude some of the doctors meet you with, when you’re going in, it’s like, ‘you’re here, there’s nothing wrong with ya’”. One individual had a similar experience with her GP when presenting with potential breast cancer symptoms, and was dismissed due to the fact that she had recently had a child:

He literally laughed at me down the phone … "you're only young. I don't think it's anything to worry about. Just put a hot cloth on your boob, you'll be fine" … I paid 30 quid for the pleasure of that … He just kept referring to my age … "You're too young to have anything wrong with you". And I was like, I don't think life works like that, it would be great if it did, but, unfortunately, it doesn't.

One participant recalled an interaction with her GP, wherein after being informed of her *BRCA* alteration status, “he was like … chill … it's no biggie … don't be too worried about it”, which she considered “about as helpful now as a slap in the head”. There were numerous counts where individuals described insensitive interactions with healthcare professionals, “one consultant … was very clinical … just asked me to open up my top and the whole team was there, and I remember crying, doing it”.

### Subtheme two: disempowerment around decision making

Participants also experienced conflicting interactions with healthcare professionals. One participant described how her physician reacted negatively to her because of her decision to proceed with screening, rather than undergo preventative surgery:

I told him that, you know, I think I was gonna keep me body parts… he agreed … then I saw him the next time he took the head off me! There was students standing outside the door when he left I said ‘I feel like I just left the principal’s office’… he was giving out hell to me that I hadn’t done anything… he said “this is a cancer clinic. People in here have cancer. You’re coming here to see if your cancer has arrived yet.

Another participant also described a stressful situation with a healthcare professional dismissing her concerns, wherein she had an active cancer diagnosis and was advised to undergo a unilateral mastectomy. This participant had, however, requested to undergo a bilateral mastectomy to reduce her cancer reoccurrence risk. This was met with resistance:

I wouldn’t like to be back there with the stress that caused… I came up against such a brick wall… I am really glad that I really found it somewhere to stand up to it…And in the end, they agreed to it. But I don’t think unless I created an awful argy bargy, that they would have done it… when I did go in for that meeting with the surgeon… I actually felt like, ‘I’m going in here to fight for my life’.

A similar issue was noted by another participant, who felt dismissed when a healthcare professional addressed her husband when he was accompanying her to an appointment to consider undergoing a salpingo-oophorectomy. She recalled how “the doctor spoke to [husband] about it. He said you do realise what this entails now, don’t you?”, and how “that was something that annoyed” her.

## Discussion

The concerns raised by the study participants indicate that the services they are engaging with are difficult to navigate. Such services are vital in mitigating a cancer risk in the *BRCA*1/2 alteration population. This uncertainty was heightened by the lack of clear information available for this population. In past research, information has been noted by *BRCA*1/2 alteration carriers as a tool that can alter the potential course of cancer development [24]. As informational needs vary depending on what healthcare services are being engaged with at any given time, it is important that relevant services and information can be revisited whenever necessary. As proposed by Dean and Fisher [21], the trajectory of uncertainty experienced by hereditary cancer cohorts will develop as the individual goes through various stages of their life. This research supports such findings, as it highlights the ongoing needs of the *BRCA*1/2 alteration population for management strategies to alleviate the disempowerment and uncertainty noted in the current study. There are avenues through which such support can be offered by the nursing profession to hereditary cancer cohorts. Psycho-educational interventions have been previously noted as efficacious in assisting in reducing anxiety in this cohort [26], and these can be tailored to ensure that the appropriate level of information is provided depending on what risk management service they are engaging with. As such, it is important to ensure that healthcare practitioners, such as nurses, are well-equipped to acknowledge and assist individuals in dealing with uncertainty.

A clear issue reported by participants in the current study was the lack of knowledge held by healthcare professionals regarding the implications of a *BRCA*1/2 alteration. There is a strong need for healthcare professionals across all specialities to be provided with assistance and training in genetics [45, 46]. Past pilot research has found that a short educational intervention delivered to public health nurses can increase knowledge and awareness of genetic disorders, alongside enhancing the motivation of nurses to learn about such conditions [47]. This is reported elsewhere, where nurses working in numerous healthcare settings have reported interest in being educated on genetics, however, this is a notably underrepresented topic in both under- and postgraduate nursing degrees [46]. This educational base is necessary to further develop the genetic knowledge of practicing nurses. In a study of 619 registered nurses, just over one quarter (27.5%) reported that they were ‘confident’ in their ability to know what information was pertinent to gauge an individual’s predisposition to developing certain conditions, such as cancer [48]. While most nurses in this study did correctly identify that family history has clinical relevance in breast (99.1%), ovarian (96.4%), and colon cancer (98%), there was a perceived deficit in their genetic knowledge. Namely, over half of the nurses (56.5%) felt they had poor or fair genetic knowledge, and out of nurses that actively saw patients (*n* = 359), only 6% reported always taking a family history [48]. This indicates that while nurses understand the importance of familial history within oncology settings, there is a lack of implementation of this into practice. Moreover, qualitative research shows that healthcare providers need to be better aware of the psychosocial issues that *BRCA*1/2 alteration carriers face, in this instance focusing on those that are young and female [49]. An acknowledgement of the widespread implications of being identified as a *BRCA*1/2 alteration carrier, and how this can impact childbearing and relationships with spouses has been highlighted as a means to support individuals in this population [49]. The current study highlighted the deficits of knowledge among healthcare professionals, as perceived by *BRCA*1/2 healthcare service users, and further bolsters the need for better education on genetics among those involved in providing care to this population.

While the results from this study demonstrated the experiences of those with a *BRCA*1/2 alteration, findings may also encapsulate the experiences of other populations with chronic conditions that are similarly living with a continued need to engage with health services. This article highlights the *BRCA*1/2 alteration populations’ need for coordinated and well-informed interactions with health care professionals. The lack of a centralised, clear cancer genetics service heightened distress in the study sample as individuals could not access relevant information. A common issue noted was poor access to services, which was often heightened due to waiting times. Additional problems emerged in trying to navigate services that were not designed with a hereditary cancer population in mind, leaving individuals often feeling as though they were a burden when presenting for appointments in symptomatic cancer units.

Contextual factors are important when addressing experiences of individuals with a *BRCA*1/2 alteration [50], and so it is important to undertake research across various countries. Furthermore, while conducted in Ireland, this is the first study to address issues in the context of a tiered healthcare system, which may reflect experiences in other similar systems internationally. The issues with waiting times within this tiered system are not unique to Ireland, and this links back to the staffing of clinical genetic services in a country (not necessarily cancer-genetic specific). A recent study investigating thirteen European countries noted that Ireland has the lowest staffing rate of clinical geneticists, non-laboratory clinical researchers and genetic counsellors, followed by Portugal and England [51]. Furthermore, this lack of service accessibility and availability heightens the divide experienced by those who can afford to pay for private care. For example, one participant depicted how they received care for managing hormones post salpingo-oophorectomy in England. This need to travel for necessary support further highlights the inequality that exists – those who are well informed and have the resources to further educate themselves can identify services that they may benefit from. If that individual has the financial capability to access these services privately, and travel, they can proactively reach out to the appropriate services. Those lacking these resources are left without and do not benefit from the additional support.

As more pathogenic variants are identified that denote a high to medium risk of lifetime breast cancer risk [52], research should further investigate the experiences of individuals from other hereditary cancer conditions. Individuals with high-risk hereditary cancer conditions such as Lynch Syndrome, or with other pathogenic variants indicating medium to low lifetime risk of developing breast cancers such as those with alterations in the *PALB*2 gene, may experience similar difficulties in accessing the appropriate healthcare in tiered systems. Similar findings pertaining to the stress created by barriers in gaining access to healthcare have already been identified in other hereditary cancer conditions. This further highlights the need for specialist cancer genetic care within the broader healthcare system [17, 53].

### Strengths and limitations

The use of a PPI panel further strengthened this research as the focus of the study was chosen based on discussion with the panel members. Furthermore, the lead author in the study carries a *BRCA*1 alteration and is heavily involved in the charitable and advocacy field for this population. The personal relationship of the researcher to the research topic and the influence of PPI in the focus of the study seemed to promote a level of openness in response to the questions, by allowing a shared ‘commonality’ between the participants and the researcher [54, 55]. This is a well reported-on phenomenon in ‘insider’ research – referring to the researcher being an active member of the population they are researching [55].

A limitation of this study design was that it was not feasible to involve the PPI panel in the analysis of this study. This was due, in part, to not having the opportunity to provide remuneration. In addition to this, there was a concern among the authors that the PPI panel may be able to identify participants from details in study transcripts. This is attributable to the small population of Ireland, and therefore relatively small population of people identified as carrying a *BRCA*1/2 alteration across the country (note that the small size of the identified *BRCA*1/2 alteration population in Ireland is likely in part due to long waiting times for genetic testing; 29). This, combined with recruitment of participants through private Facebook groups, led the researchers to conclude that the risk of identifying participants by PPI panel members was too high, should they be involved in analysis. Themes were therefore reviewed by the study supervisor (AMG), with continued reflexive practice employed. Informal discussions with members of the *BRCA*1/2 alteration population also took place, to report results and gain insight into the comprehensibility of themes. This was not included as official PPI activity since it was neither formally structured nor planned. It did, however, influence the interpretation of study findings. Through these discussions of developed themes, NW was afforded the opportunity to further reflect on how the community at large interpreted results. The inaccessibility of healthcare was especially prominent in discussions, which bolstered the meaning derived from quotes for the subtheme “Healthcare Services as Largely Inaccessible”. While the lack of formal input into analysis was a potential limitation, the significant contribution from the Irish *BRCA*1/2 alteration community at the outset of this research ensured that the study aims, and design, were deemed of relevance to the population under study.

Alongside this, the familiarity of the topic at hand to the researcher was an aspect that required continued addressing throughout the data analysis. In reflexive thematic analysis, the subjectivity of the researcher is not considered a threat to the study findings, nor a negative source of bias [38]. Rather, it is viewed as a tool through which understanding of the data is developed. With this study led by a member of the Irish *BRCA*1/2 alteration community (NW), a considerable amount of time was spent reflecting on how NW interpreted and situated their understanding of the data produced. In research of this nature, there is potential for an inside researcher to highlight shared experiences with participants, over experiences not shared [55]. From the outset of this research, NW was cognisant that the current study should go beyond their own experience. Therefore, substantial time was spent on ensuring that participants’ experiences were centred in the study analysis. This was acknowledged throughout reflexive practice and discussed regularly in debrief with the research supervisor (AMG). Through this, NW was able to differentiate between personal experiences and those discussed by the participants of the study. This ensured that the subjective experiences of NW were acknowledged during the development of themes, while also ensuring that the final iteration of themes represented the individual experiences of the study participants. This was further strengthened by maintaining a compassionate distance with participants. This refers to being aware of the role that one plays within a conversation, through acknowledging that while the experiences of *BRCA*1/2 alterations may be mutual, the emphasis of the interview is not to find shared commonalities between researcher and participant, but to hear about theirs. In future, including the PPI panel in the analysis of the study findings may further enhance this process.

A potential limitation of this study was participants were not selected based on time since the identification of their *BRCA*1/2 alteration, nor were participants recruited on the basis of preventative measures undertaken. This was mainly due to practical considerations – as minimal research has been conducted in this population to date, the researchers left recruitment as open as possible. Furthermore, all the PPI panel sampled in this research identified as female, and all PPI panel members and the majority of participants were White-Irish. This is not representative of Ireland’s population, a country with a growing rate of non-native residents, whereby in 2018 over 12% of the population were non-Irish nationals [56]. Populations presenting for genetic testing tend to be predominantly non-immigrants, with higher rates of education [57]. A recent systematic review investigating barriers to accessing genetic testing for minority groups reported lower knowledge about, and awareness of, the need for cancer genetic testing [58]. It is, therefore, important to include minority groups in research of this kind. In the Irish Travelling community, for example, genetic variants associated with cancer susceptibility have been noted, namely *BRCA*2 alterations [59]. This population are known to experience disparities in both access to and quality of healthcare and health related information [60]. Future research should ensure that the implications of being a member of minority groups on access to and engagement with genetic healthcare services are examined.

## Conclusion

This study highlights the undue burden that individuals with a *BRCA*1/2 alteration face when trying to navigate a medical system which was not designed with a hereditary cancer population in mind. It notes the importance placed by the *BRCA*1/2 population on the quality of both available services and interactions with healthcare professionals. Due to underdeveloped services and a lack of access to appropriate services, hereditary cancer populations continue to experience uncertainty. While the provision of preventative care for the management of hereditary cancer risks is still relatively novel, the findings from this study suggest that the perceived quality and availability of care may play a role in the adjustment experiences of the *BRCA*1/2 population. Such populations should be enrolled in a healthcare system that is well-equipped and designed to facilitate individuals with hereditary cancer conditions.

## Supplementary Information

**Additional file 1.**

## Data Availability

The datasets generated and/or analysed during the current study are not publicly available due to the sensitive nature of the questions asked in this study. Respondents were assured raw data would remain confidential and would not be shared, as participants would be easily identifiable from the details provided. As such, research data are not available as participants did not provide explicit consent for this.

## Abbreviation

*BRCA*1/2: Breast Cancer Gene 1, Breast Cancer Gene 2
