# Supplementary material for: A qualitative reflexive thematic analysis into the experiences of being identified with a BRCA1/2 gene alteration: “So many little, little traumas could have been avoided”
Source: BMC Health Serv Res. 2022 Aug 6;22:1007. doi: 10.1186/s12913-022-08372-w (PMC9357316; doi:10.1186/s12913-022-08372-w)
Supplement: Supplementary file 1 — Additional file 1. [file 12913_2022_8372_MOESM1_ESM.docx]

| Supplementary Table 1.  Standards for Reporting Qualitative Research. | | |
| --- | --- | --- |
| No. | **Topic.** | **Page Number.** |
| Title and abstract | | |
| S1 | Title | 1 |
| S2 | Abstract | 1 |
| Introduction | | |
| S3 | Problem formulation | 2 – 5 |
| S4 | Purpose or research question | 4 |
| Methods | | |
| S5 | Qualitative approach and research paradigm | 7 |
| S6 | Researcher characteristics and reflexivity | 5 – 6 |
| S7 | Context | 5 – 6 |
| S8 | Sampling strategy | 6 |
| S9 | Ethical issues pertaining to human subjects | 6 |
| S10 | Data collection methods | 6 – 7 |
| S11 | Data collection instruments and technologies | 7 |
| S12 | Units of study | 8 |
| S13 | Data processing | 6 – 7 |
| S14 | Data analysis | 8 |
| S15 | Techniques to enhance trustworthiness | 7 – 8 |
| Results/findings | | |
| S16 | Synthesis and interpretation | 9 – 17 |
| S17 | Links to empirical data | 9 – 17 |
| Discussion | | |
| S18 | Integration with prior work, implications, transferability, and contribution(s) to the field | 17 – 21 |
| S19 | Limitations | 20 – 21 |
| Other | | |
| S20 | Conflicts of interest | 22 |
| S21 | Funding | 22 |

| Supplementary Table 2.  GRIPP2 Short Form | | |  |
| --- | --- | --- | --- |
| Section and Topic | **Item** | **Reported on page No** | |
| 1: Aim | Report the aim of PPI in the study | | p. 5 |
| 2: Methods | Provide a clear description of the methods used for PPI in the study | | p. 6 – 7 |
| 3: Study results | Outcomes—Report the results of PPI in the study, including both positive and negative outcomes | | p. 11 |
| 4: Discussion and conclusions | Outcomes—Comment on the extent to which PPI influenced the study overall. Describe positive and negative effects | | p. 23 |
| 5: Reflections/critical perspective | Comment critically on the study, reflecting on the things that went well and those that did not, so others can learn from this experience | | p. 23 |

Supplementary Figure 1. Themes identified in the data.
